# Multi-Level Neuromorphic Devices Built on Emerging Ferroic Materials: A Review

**DOI:** 10.3389/fnins.2021.661667

**Published:** 2021-04-28

**Authors:** Cheng Wang, Amogh Agrawal, Eunseon Yu, Kaushik Roy

**Affiliations:** School of Electrical and Computer Engineering, Purdue University, West Lafayette, IN, United States

**Keywords:** neuromorphic, multi-level, spintronic, ferroelectric, device, computing

## Abstract

Achieving multi-level devices is crucial to efficiently emulate key bio-plausible functionalities such as synaptic plasticity and neuronal activity, and has become an important aspect of neuromorphic hardware development. In this review article, we focus on various ferromagnetic (FM) and ferroelectric (FE) devices capable of representing multiple states, and discuss the usage of such multi-level devices for implementing neuromorphic functionalities. We will elaborate that the analog-like resistive states in ferromagnetic or ferroelectric thin films are due to the non-coherent multi-domain switching dynamics, which is fundamentally different from most memristive materials involving electroforming processes or significant ion motion. Both device fundamentals related to the mechanism of introducing multilevel states and exemplary implementations of neural functionalities built on various device structures are highlighted. In light of the non-destructive nature and the relatively simple physical process of multi-domain switching, we envision that ferroic-based multi-state devices provide an alternative pathway toward energy efficient implementation of neuro-inspired computing hardware with potential advantages of high endurance and controllability.

## 1. Introduction

The recent advancements of data-driven learning paradigm such as artificial deep neural networks (DNN) have achieved superhuman performance in various applications including image/pattern recognition, natural language processing, and developing autonomous intelligence (LeCun et al., 2015). However, the energy consumption of artificial intelligence (AI) implemented in today's computers is significantly higher compared to that of a human brain (Cox and Dean, 2014). The energy inefficiency of such hardware is largely attributed to the von-Neumann memory bottleneck due to the separation of memory and compute units and the limited on-chip memory density in computing hardware. For instance, DNNs are usually implemented in graphic processing units (GPUs), which desire large area and power consumption in presence of growing DNN model sizes and large amount of data to process. Neuromorphic computing is an emerging computing paradigm that aims for building bio-plausible computing systems in pursuit of brain-level efficiency in cognitive processing (Mead, 1990; Roy et al., 2019). Recently, remarkable implementations of neuromorphic hardware such as TrueNorth (Merolla et al., 2014) and Loihi (Davies et al., 2018) have been demonstrated based on complementary metal-oxide-semiconductor (CMOS) technologies. But CMOS-based technologies require large number of transistors to implement neuronal and synaptic functions, leading to increased cost of energy and area.

**Figure 1:**
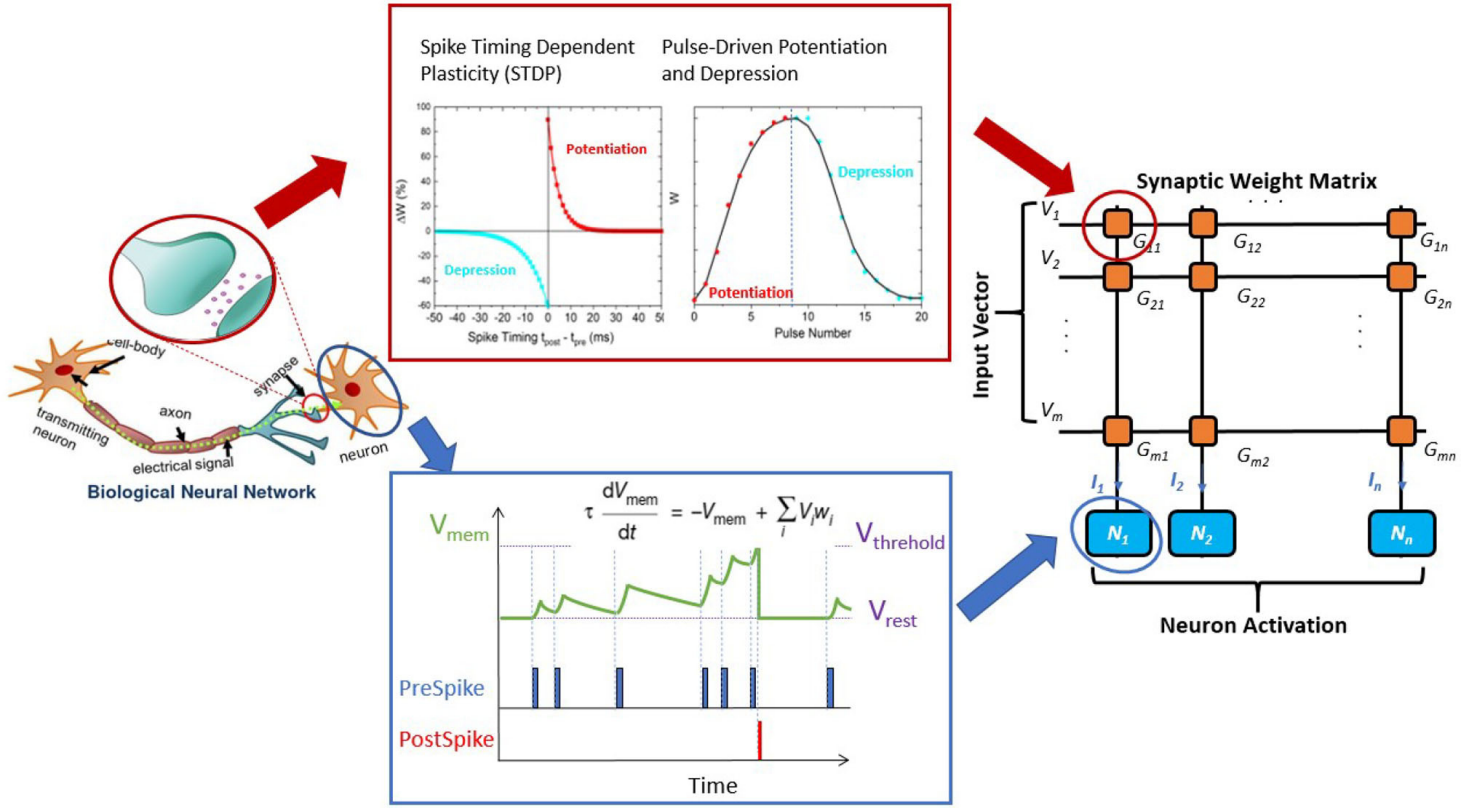
Concept of a biological neural network and its hardware implementation in crossbar arrays. Left panel illustrates that two biological neurons are interconnected by a synapse. The strength of the synaptic connections (synaptic weights) can be modified depending on the relationship between the two neurons. A crossbar array implementing artificial neural networks containing neurons connected by synaptic devices is shown in the right panel. The center panel describes synapse **(top)** and neuron **(bottom)** models. The activation of a neuron is controlled by its membrane potential, and its dynamics can be described of a leaky integrate-fire (LIF) neuron model. An accurate description of the membrane potential desires devices that can represent analog state values. Neurons are interconnected by synapses, which can be put into crossbar devices with variable conductance states. Spiking timing dependence plasticity (STDP) and pulse driven potentiation/depression of a synapse is shown to illustrate one bio-plausible learning mechanism based on synaptic plasticity.

On the other hand, emerging non-volatile memories (NVM) based on novel physical mechanisms can lead to significant reduction of leakage power and achieve high on-chip density compared to CMOS, while mimicking key neuro-synaptic functionalities for neuromorphic computing (Yu and Chen, 2016; Li et al., 2019). Particularly, the emerging NVM technologies have great potential to provide scalable and energy efficient building blocks for crossbar based in-memory computing by performing computation within memory arrays (Ambrogio et al., 2018; Ielmini and Wong, 2018). With crossbar computing, the product of an input vector (voltage) and a weight matrix (conductance) can be obtained from the accumulated output currents, following Ohm's Law. Such configuration leads to efficient hardware realization of matrix-vector multiplication (MVM) operations, which are ubiquitous in both bio-plausible computing and standard DNN models. Therefore, enabling crossbar computing will not only facilitate the development of neuromorphic fabrics, but also improve hardware efficiency of executing generic AI/machine learning algorithms. As for neuromorphic processing, several mathematical models have been proposed to describe the neuronal models and synaptic learning rules of biological nervous system (Hodgkin and Huxley, 1952; Izhikevich, 2003), laying the foundation for neuro-mimetic implementation in hardware. Figure 1 shows a hardware-implemented neural network with exemplary leaky-integrate and fire (LIF) neurons and synapses based on spike timing-dependent plasticity (STDP) or pulse-driven synaptic learning rules (Yu et al., 2019). Emulation of both neurons and synapses have been experimentally demonstrated using emerging NVMs (Tuma et al., 2016; Islam et al., 2019).

In order to provide high density on-chip memory as well as efficient emulation of synaptic plasticity and neuron activations, it is desirable to have programmable multi-level NVM devices. The capability of multiple states per device will not only enlarge the capacity and precision of synaptic weight storage in neuro-inspired computing, but also lead to benefits in generic memory applications due to increased density. Furthermore, multi-level NVM devices can realize both emulation of the aforementioned bio-plausible neurons (Sharad et al., 2013; Burr et al., 2017) and in-device implementation of various analog neuron models such as (shifted) sigmoid (Siddiqui et al., 2019) and rectified linear units (ReLu) (Lashkare et al., 2018), which are frequently used in DNNs. Among the emerging NVMs, resistive random-access memory (ReRAM) (Hu et al., 2014) and phase change memory (PCM) (Boybat et al., 2018) can provide high memory density as well as multi-level cells. However, the electroforming process involving ion motion of ReRAM and the melting-crystallization process of PCM induce endurance and reliability issues: large variations among devices and sizeable drifts over time (Eryilmaz et al., 2015). The low endurance significantly limits the numbers of writes, preventing the use of ReRAM and PCM for training large-scale AI models. The large device variation not only makes it difficult to program such NVM devices to a desirable conductance states but also places challenges to differentiate and sense the multiple levels. Moreover, due to the large conductance drift over time in PCM, erroneous results may occur even for inference-only tasks when running a pre-trained model mapped in PCM crossbar arrays. At present, although various types of NVM devices have been proposed and studied, it is still challenging to provide a reliable, scalable, and energy efficient hardware solution for multi-level neuro-mimetic devices (Burr et al., 2017; Schuman et al., 2017; Yan et al., 2018; Chakraborty et al., 2020b; Kim et al., 2020).

In contrast, devices using ferroic (magnetic and ferroelectric) materials such as magnetic RAM (MRAM) (Bhatti et al., 2017) and ferroelectric RAM (FeRAM) (Ishiwara, 2012) can provide better endurance and more energy-efficient writing, leveraging the unique properties of spin or charge polarization. In particular, the spintronic materials are promising for high endurance due to the absence of physical ionic motion in magnetization switching, while ferroelectric field-effect transistors (FeFET) could offer superior CMOS compatibility. While spintronic (magnetic) and ferroelectric materials have been traditionally investigated for binary memory, there has been growing interest in exploiting them for multi-level neuromorphic devices for functionalities such as synaptic plasticity and membrane potential modulation in neurons. More interestingly, the switching mechanisms and memory effects of ferroic materials share remarkable resemblance with biological neural systems, suggesting a possible path of developing bio-plausible hardware primitives. However, although the NVM devices based on ferroic materials might have larger cell areas than ReRAM and PCM, they can still achieve better density compared to CMOS.

In this review article, we explore ferroic materials as possible material of choice for neuromorphic with multi-level resistive devices. It is shown that spintronic and ferroelectric materials leveraging multi-domain switching dynamics enable devices to obtain multi-level states with improved controllability and endurance compared to ReRAM and PCM. The rest of this article is organized as follows. Section 2 focuses on spintronic devices, where both domain wall (DW) motion in ferromagnets and multi-domains induced in exchange-coupled heterostructures can be leveraged to achieve multi-level nanometer-scale devices. Section 3 focuses on ferroelectric devices, including FeFET and ferroelectric tunnel junctions (FTJ) as the basic configurations for generating ferroelectric multi-level states. Both fundamental material physics of multi-level devices as well as representative demonstrations of neural functionalities are covered. Section 4 reiterates the opportunities with ferroic multi-level devices toward developing neuromorphic hardware in comparison with other technologies. We will highlight key advantages and challenges for each of these technologies, followed by discussions and proposals regarding the potential pathways for addressing the challenges. We conclude this review with the proposal that through the co-design of device, circuit, and algorithm, multi-level ferroic devices could provide exciting opportunities of constructing large-scale and energy efficient cognitive computing systems.

## 2. Spintronic Devices

**Figure 2:**
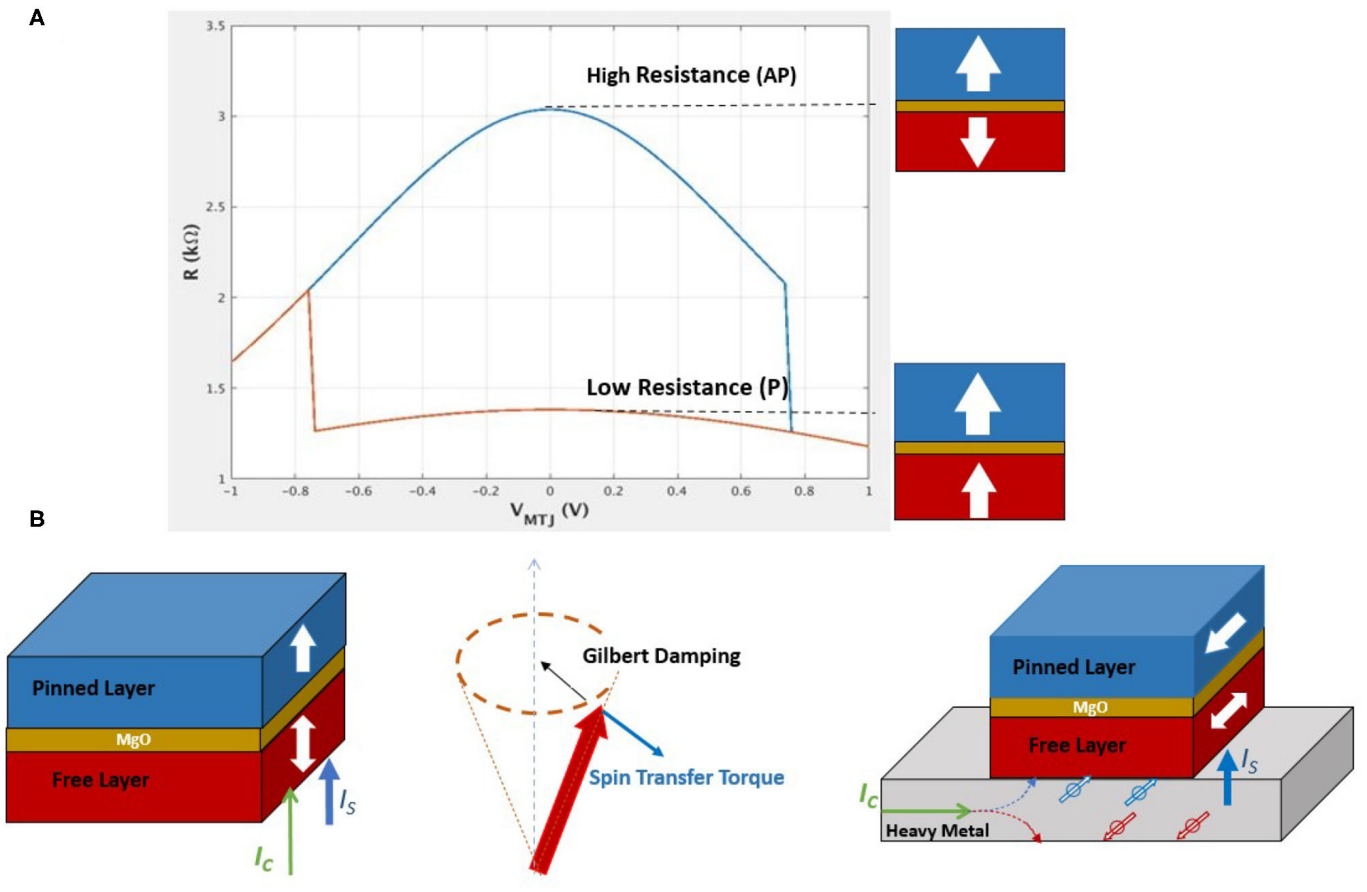
**(A)** Magnetic tunnel junction (MTJ) and tunneling magnetoresistance (TMR). Resistance of MTJ depends on the relative orientations of the two ferromagnetic layers next to the tunnel barrier. High/low resistance states correspond to anti-parallel (AP)/parallel (P) configuration of the magnetic ordering in the free layer and reference layer. **(B)** Spin transfer torque (STT) and spin-orbit torque (SOT). STT is originated from spin polarized current going through an MTJ. The STT effectively switches spins by countering against the Gilbert damping of the free layer magnetic moments. SOT is a result of spin Hall effect at interface of ferromagnetic/heavy metal layers, where a charge current flowing along the heavy metal layer can induce a transverse spin current flowing into the adjacent ferromagnetic layer.

Spintronic materials have shown clear advantages for developing next generation non-volatile memory with potential combination of high speed, low power, and unparalleled endurance. In particular, magnetic tunnel junctions (MTJ) have been extensively investigated and demonstrated reliable memory read and write schemes in device dimension down to tens of nanometers (Ikegawa et al., 2020). As is illustrated in Figure 2, a MTJ in MRAM memory cell comprises of two FM layer separated by a thin tunnel barrier. Conductance of MTJ under applied voltages will be high (low) when the magnetizations in the two ferromagnetic layers are parallel (anti-parallel) due to spin-dependent tunneling across the barrier (Parkin et al., 2004). In practical MTJ device stack, one of the FM layer is pinned by additional structures forming a reference layer (RL), while the other FM layer defined as free layer (FL) can be switched between the two states under external stimulus including external magnetic field, or spin-polarized current induced spin transfer torque (STT) (Slonczewski, 1996; Diao et al., 2007) and spin-orbit torque (SOT) (Liu et al., 2012), as illustrated in Figure 2B.

While MTJs can naturally store binary information with high accuracy and thermal stability based on the bi-directional magnetizations, having controllable multilevels in the FL is more of interest for device level emulations of neuromorphic functionalities. Note that ferromagnetic materials are known to maintain long-range magnetic ordering with long retention and high stability against perturbations due to the strong exchange interactions among localized magnetic moments therein. Conversely, introducing stable multi-domain configurations in magnetic-based materials inevitably becomes difficult thanks to the need of countering the forming of long-range magnetic ordering, imperatively urging new mechanisms from material and device structure level for inducing multi-domains functionalities. In the following subsections, various approaches introducing multi-level devices are highlighted and some prototypes of demonstrating brain-inspired computing are discussed.

### 2.1. Multi-Level Spintronic Devices Based on Domain Wall Motion

A natural path to multilevels in spintronics is to split the single-domain magnetizations as formed in MRAM devices to multiple domains (Fong et al., 2016). It is known that switching of magnetic thin films can involve mechanisms of nucleation formation and domain propagation, suggesting a possibility to generate intermediate multi-domain of magnetic textures between the bi-stable states. In order to have stable configurations of multiple domains in continuous ferromagnetic thin films, additional mechanisms are required to maintain the pinning of domain walls between spin-up and spin-down regions. The DWs can be pinned or displaced, depending on the combined effects of material properties such as exchange coupling among magnetic moments, shape anisotropy determined by device geometry, as well as local defects (Beach et al., 2006; Thomas et al., 2006; Emori et al., 2013). Conceptually, such domain wall motion (DWM) in a ferromagnetic thin film can generate a near-continuous variation of magnetic states from one direction to the other, resulting in variable resistance states of a magneto-resistive device described by a model with parallel resistors.

#### 2.1.1. Device Fundamentals of Domain Wall Motion

**Figure 3:**
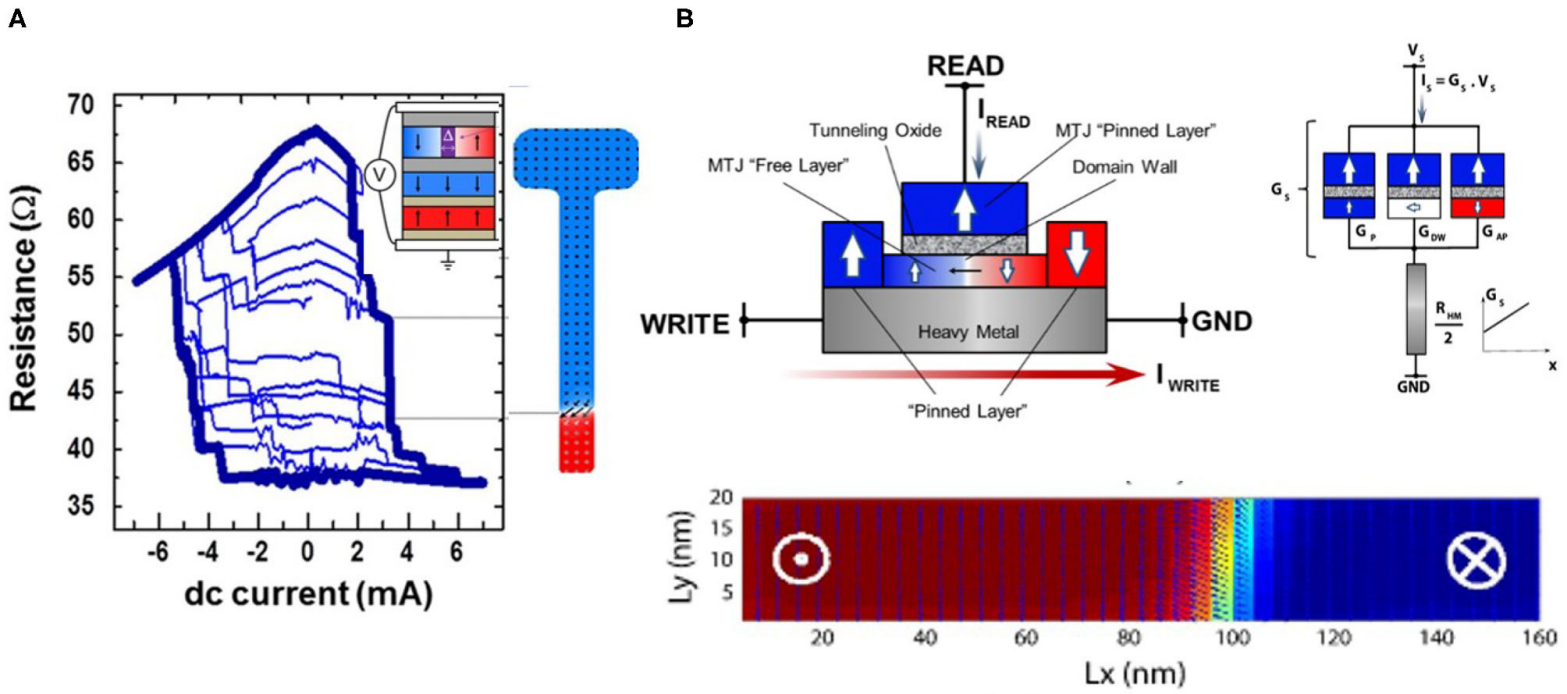
Domain wall motion (DWM) based multi-level devices. **(A)** STT-driven DWM in a MTJ-based device. Lequeux et al. Scientific Reports 6, 31510 (2016), Copyright 2016 Author(s), licensed under a Creative Commons Attribution (CC BY) license. **(B)** SOT-driven DWM device. Dzyaloshinskii-Moriya exchange interaction across the heavy metal and ferromagnetic layer provides effective magnetic fields that ensure deterministic switching. Reproduced with permission from Sengupta et al. IEEE Transactions on Biomedical Circuits and Systems, 10, 1152–1160 (2016), Copyright 2016 IEEE.

The idea of using DWM driven by current-induced torque has sparkled a plethora of studies built on the mostly matured STT-switching technology (Wang et al., 2009; Lequeux et al., 2016). It has been proposed and demonstrated that with special engineering of device geometry, non-volatile multi-level resistance states can be realized in an MTJ with perpendicular magnetic anisotropy (PMA). As is shown in Figure 3A, a DW in the free layer of MTJ with the shape of an elongated stripe can be displaced by applied electric currents, leading to modifications in MTJ resistance following the relationship:

(1) $$\begin{array}{l} G\left(x\right)=G_{P}*x/L+G_{AP}*\left(1-x/L\right)+G_{DW} \end{array}$$

where G_P_ and G_AP_ are the parallel and antiparallel conductance respectively, and x is the domain wall position in a stripe of length L. STT-DWM is controlled by the magnitude and polarity of the spin polarized currents across the FL. The current-driven DWM device can potentially work as a two-terminal compact device following the STT-MRAM configuration. Furthermore, it was recently found that in magnetic heterostructures such as oxide/ferromagnetic/heavy metal stacks, a chiral DW with Néel configuration can be formed and stabilized in perpendicularly magnetized thin films due to Dzyaloshinskii-Moriya exchange interaction (DMI) at the FM/HM interface and the broken inversion symmetry in the heterostructure stack (Emori et al., 2013). It is observed that the Néel Wall can be efficiently driven by the spin orbit torque (SOT) originated from spin Hall effect (SHE) of the heavy metal layer, as is shown in Figure 3B. Therefore, DWM driven by SOT in a MTJ/HM heterostructures can be leveraged for programming the multi-level device conductance (Sengupta et al., 2016b). While in general a magnetic field is needed for deterministic SOT switching of PMA materials due to the in-plane spin polarization (Liu et al., 2012), it is found that the interfacial DMI could effectively provide the desired magnetic field toward field-free SOT driven DWM (Emori et al., 2013). Current-driven DW motion in heavy-metal/ferromagnet/oxide structures is naturally explained by the combination of the SHE and DMI. The SHE produces the sole current-induced torque, and DMI stabilizes chiral DWs while permitting uniform DW motion with very high efficiency. The writing speed of DWM-SOT devices is characterized by the domain wall velocities, which increases with the current density up to saturation in absence of pinning sites. Although domain wall velocity can be as high as 10^2^ m/s (Beach et al., 2006; Agrawal and Roy, 2018), in practice DW motion can be hindered due to pinning in magnetic thin films, making it challenging to have precise control and efficient manipulation of DWs (Thomas et al., 2006). The readout mechanism in the proposed device is similar to that of a STT-MRAM memory cell, while the SOT is generated from a lateral charge currents which has a separated flow path from the reading operation across MTJ stack. The major advantage of the three-terminal device is the decoupled read and write paths which will eliminate the read disturbance issue of MTJ and thus lead to significant improvement in device endurance. Recently it is confirmed experimentally that SOT-DWM can be used for artificial synaptic devices in MgO/CoFeB/Ta heterostructures. In the following subsection, various prototypes implementing neuromorphic functionalities based on DWM spintronic devices are discussed. Although we focus on SOT-driven configurations given the advantage of separate read/write paths, similar mechanism will also work for STT-driven configurations.

#### 2.1.2. Domain Wall Motion Based Neuromorphic Devices

**Figure 4:**
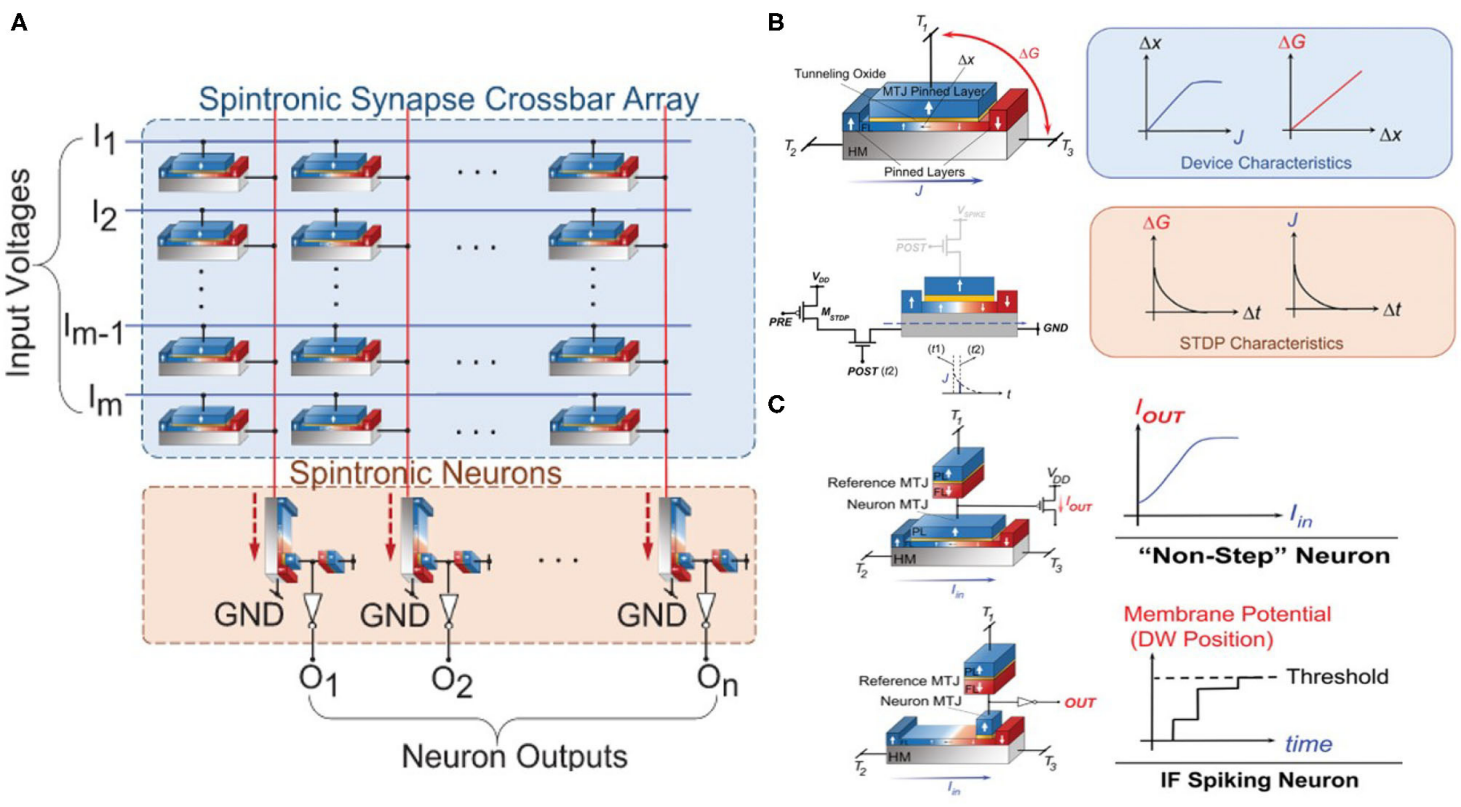
**(A)** All-spin crossbar array with both synapse and neurons based on SOT-DWM devices. **(B)** DWM-based spintronic synaptic devices. **(C)** DWM-based analog and IF spiking neurons. Figures reproduced with permission from Sengupta and Roy, Appl. Phys. Rev. 4, 041105 (2017). Copyright 2017 AIP Publishing.

A direct application of SOT-driven DWM device will be crossbar implementation for MVM computing engine. Leveraging a linear dependence of conductance on the DW position which is subsequently linearly dependent on the driving electric current (before saturation), SOT-DWM devices in a crossbar array can have their DW position (and thus conductance states) accurately programmed to map a synaptic weight matrix, as is illustrated in Figure 4 (Sengupta et al., 2016b; Sengupta and Roy, 2017). Parallel dot product of vector (voltage) and matrix (conductance) can be directly executed following the Kirchoff Current Law. Given the non-volatility of conductance states in those multi-domain devices, we could just set the devices once with pre-trained weight matrix and reuse the stored weights during inference, eliminating additional memory access and data transfer. The advantage of separated read and write paths in SOT-DWM is evidently demonstrated, as the MVM operations at inference only involves reading path and thus read disturbance to the states is minimized. The DWM-based multi-level device can also implement STDP, another important synaptic characteristics. With STDP learning rule, presynaptic spike arrival before the occurrence of postsynaptic spike leads to long-term potentiation (LTP) of the connecting synapse, whereas spike arrival after postsynaptic spike leads to long-term depression (LTD) of the same synapse. The magnitude of the relative change in synaptic strength (ΔW) decreases exponentially with the timing difference between the preneuron and postneuron spikes. A key step to realize STDP with a DWM-based spin device is to link the timing of the pre-neuron and post-neuron to the conductance change in the interconnected SOT-driven DWM synapse. One approach as illustrated in Figure 4 is to provide exponential variation of HM currents modulated by the timing of PRE and POST neurons in circuit, assuming that the DW displacement and the device conductance change is linearly dependent on the magnitude of the HM current. By biasing the interfaced transistor M_STDP_ in the sub-threshold regime, current flowing through the transistor will vary exponentially with the gate voltage. For instance, in the case of LTP, the turning ON of “POST signal” gate combined with a linear increase of the “PRE signal” gate voltage, will lead to an exponentially varying programming current connecting the PRE and POST gates, depending on the timing window between PRE and POST signals. In presence of increases in the spike timing difference, the M_STDP_ driven from cut-off to the sub-threshold region will decrease the HM current and thus the resultant conductance change ΔG exponentially. In order to ensure the programmability of DWM and resolution of STDP, it is required that the rise time of the M_STDP_'s gate voltage is much longer than the post-spiking duration. In the proposed configurations, the M_STDP_ transistor with μs rise time of gate voltage and ns spiking duration were used (Sengupta et al., 2016a). The capability of synaptic weight storage as well as STDP with simple peripheral circuits makes spintronic based hardware promising for emulating complex brain-like algorithms.

In addition to synaptic plasticity, neuronal behaviors can also be realized based on the domain dynamics involving SOT-driven DWM. While a mono-domain SOT-MTJ or STT-MTJ is fully capable of making a stochastic spiking neuron based on the sigmoidal switching probability function of excitation currents (Sengupta et al., 2018), being able to generate intermediate state in device could enhance the versatility of spintronic neurons and greatly extend the capability of emulating complex neuron functionalities. Following the multi-domain magnetic configuration in a SOT-DWM device, an analog nueron can be implemented following a similar device structure of the synaptic device discussed above. Such analog devices with almost continuous output values can directly mimic the behaviors of saturating rectified linear units (saturating ReLU), or sigmoidal neurons which are predominantly applied in state-of-art deep artificial neural networks (Sengupta and Roy, 2017). Moreover, SOT-driven DWM could also utilize variations of positions in device to mimic IF neuron, which is an essential building block for hardware implementation of spiking neural networks. As is shown in the lower panel of Figure 4C, a DW located in the FL away from the MTJ sensing region may be pushed toward the MTJ region by applied current, in analogy to an increase of membrane potential due to accumulated intake of excitation. As soon as the DW enters the sensing area under the tunnel barrier, resistance change will be sensed following the magnetization switching due to DW motion and subsequently the output terminal will generate a spike. Therefore, displacement of DW positions enables the representation of changing membrane potentials in biological neurons, providing an increased level of bio-fidelity compared to a binary stepped neuron using single domain MTJ.

#### 2.1.3. Prospect of Domain Wall Based Multi-Level Devices

Compared to other memristive technologies, spintronic DWM devices provide a feasible means of leveraging material physics for low-power and high-endurance implementations of neuromorphic functionalities. In particular, the SOT-driven DWM device configuration enables realization of an all-spin neuromorphic computing block including both synapse and neuron units, while the low operation voltages as well as separated read and write paths at device level may lend further advantages. At present, DWM mechanism is one of the most investigated feature for developing spin-based neuromorphic devices. While huge potential of DWM devices has been demonstrated in both simulations and experiments, there are still challenges to address toward large scale practical implementation of such technology. For example, most DWM devices relies on precise control and sensing of a single domain wall, which often needs a quasi-1D device shape for confinement of the domain dynamics. Such constraint on device geometry lead to large footprints along the DW propagation direction (ranging from 1 to 10 μm), and thus hindering the deployment of the DWM devices beyond prototype demos (Cai et al., 2017; Jin et al., 2019; Siddiqui et al., 2019). Novel ideas such as introducing skyirmions have been investigated (Chen et al., 2018), potentially extending the current single wall based devices to multiple DW configuration (Song et al., 2020), but more work is needed to illustrate a viable path of scalability and controllability using skyrmions. Moreover, the assumption that DW displacement is linearly dependent on applied currents may not always hold in practice, due to the presence of random local device/material defects that may trigger irregular pinning/depinning, leading to erroneous results in real devices. These challenges in DWM devices also motivate the community in search of material-level mechanism beyond fully relying on motion of a single domain wall. As is discussed in the following subsection, multi-state devices built on exchange-coupled heterostructures could address some of these concerns and may pave a promising pathway for building scalable neuromorphic primitives.

### 2.2. Multi-Level Spin Devices Based on Exchange-Coupled Systems

In this section, we will focus on two categories of approach based on magnetic exchange-coupled heterostructures. The first type relies on antiferromagnetic (AF) ordering, and particularly its interaction with ferromagnetic ordering to introduce non-coherent response to external excitation such as spin currents. The idea of using AF materials to modify FM in bilayer F/AF blocks has already been used such as exchange bias in MTJ stack used in magnetic sensor and MRAM (Parkin et al., 1999), while the adoption of AF for neuro-inspired device level granularity is emerging very recently (Fukami et al., 2016). The other type is to introduce micro-structure modifications into magnetic thin films in order to facilitate the divisions of magnetic domains with the help from material segregation. While the underlying technique of fabricating continuous/granular exchange coupled composites has been successfully implemented for boosting the storage density of binary magnetic storage over the past decade, its potential adoption for neuromorphic applications is only recently proposed (Wang et al., 2019). We will discuss the basic material selection and proposed device structure, followed by highlights of recent demonstrations of realizing neuro-inspired functionalities such as memristive synapses.

#### 2.2.1. Material Physics of Exchange-Coupled Systems

**Figure 5:**
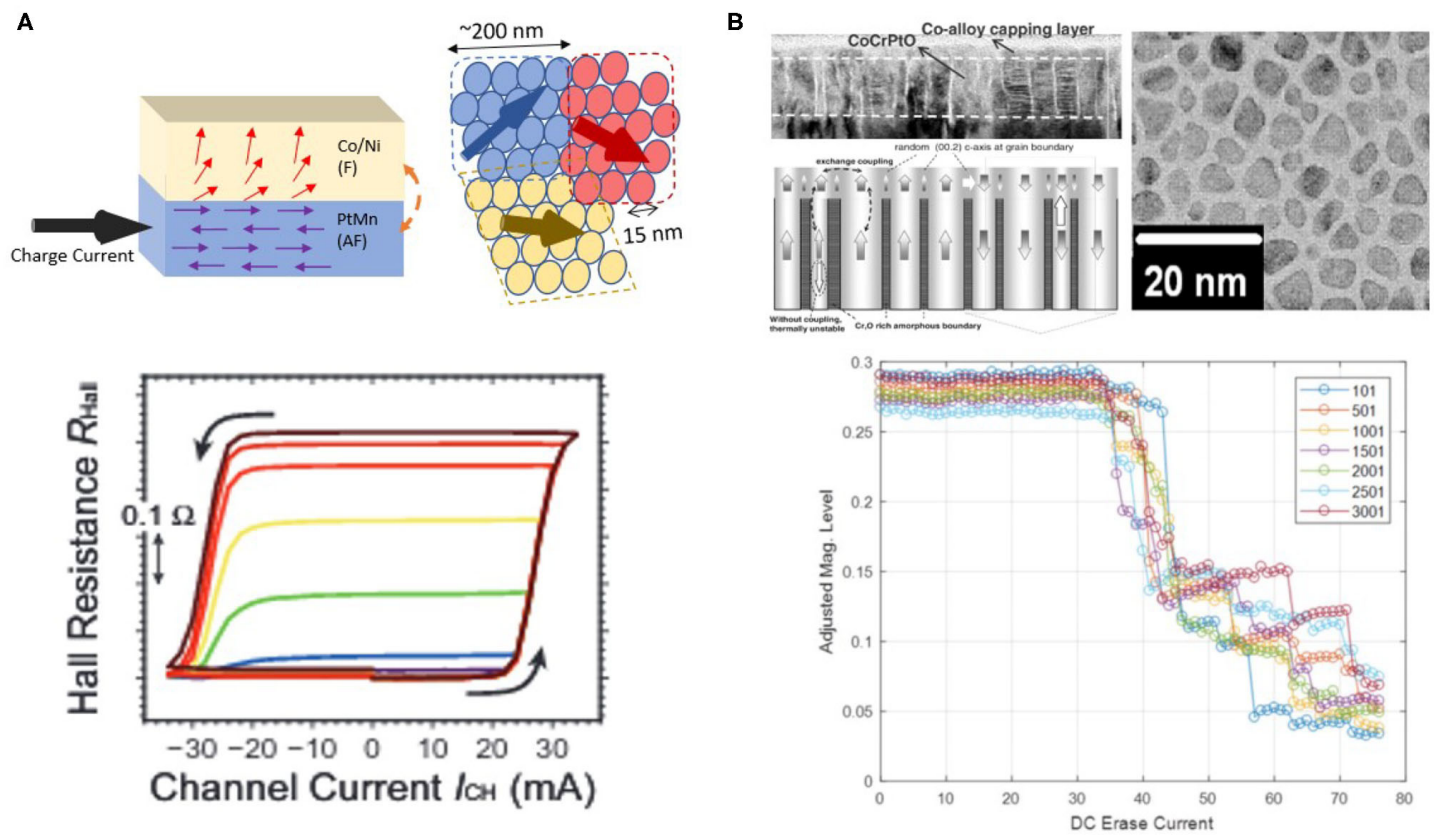
**(A)** Exchange-coupled F/AF bilayer structures with multi-level states. The top panel illustrates the bilayer structure and magnetic configurations, while the bottom panel shows multi-level Hall resistance and hysteresis loop. Figure adapted with permission from Borders et al. Appl. Phys. Express 10 013007 (2017). **(B)** Exchange-coupled continuous-granular structures with multi-level states. The top panel shows the material structure of the continuous/granular composite, while the bottom panel shows multi-state magnetization under an increasing writing field. Figure adapted with permissions from Choe et al. IEEE Trans. Mag., 41, 3172–3174 (2005) Copyright 2005 IEEE, Tham et al. IEEE Trans. Mags. 43, 671–675 (2007) Copyright 2007 IEEE, and Wang et al. U.S. Patent Application No. 16/255,698.

AF materials, which by definition have local spins ordered in compensated patterns (e.g., anti-parallel with neighboring spins), can facilitate multi-domains due to the absence of long range exchange interaction and dipole fields. In polycrystalline AF metallic thin films such as PtMn or IrMn, AF grains are formed with a dispersion of crytalline orientation and grain size, leading to variation in the AF ordering orientation and distribution of switching energy barrier among AF grains. Therefore, an inhomogeneous exchange bias is expected at a interface of FM/AF bilayer heterostructure. In presence of external fields or spin currents, the nucleation of the different regions in the FM layer may be impacted differently by the adjacent AF domains underneath, leading to multiple domains with non-coherent nucleation or gradual switching of the whole device area characterized by sloped hysteresis curves (Fukami et al., 2016). By replacing non-magnetic heavy metal with AF such as PtMn, a perpendicularly magnetized FM can be switched by SOT in a analog fashion, suggesting an exciting potential of integrating into practical SOT-MTJ devices, as is shown in Figure 5A. Meanwhile, it is reported that the multi-domain behavior will vanish when device dimension reduced to about 200 nm (Kurenkov et al., 2017), although the physical AF grains are typically as small as 15 nm. This observation suggests that magnetic cluster size in the continuous FM layer remains significantly larger than the scale of grain size, even under an inhomogeneous exchange bias from the AF grains. It remains challenging to find a feasible way so that adjacent AF can induce FM domains down to the size of AF grains. Interestingly, devices built with AF-only materials recently have also demonstrated multi-level resistance states, though more work is needed to search for better sensing mechanism of AF order without assistance of FM (Olejník et al., 2017).

In addition to bringing in F/AF exchange interaction from polycrystalline AF layer, interlayer exchange coupling between two ferromagnetic layers can also lead to effective splitting of the ferromagnetic order, if microstructure modifications can be introduced into one of the two ferromagnets. In pursuit of high density data storage, such technique has been matured and successfully implemented as exchange coupled composite medium in the magnetic recording industry. As is shown in Figure 5B, state of the art storage medium can have perpendicularly magnetized alloys (such as CoPt) grow in columnar structures with grain size averaged about 7–8 nm (Choe et al., 2005; Tham et al., 2007). More importantly, the intergranular coupling between the ferromagnetic columns can be greatly suppressed by the non-magnetic segregating oxide material (such as SiO_2_ and TiO_2_), leading to a magnetic cluster size similar to the physical grain size. The grains with a finite switching field distribution will respond to external excitation non-coherently, generating multi-domain states. It has been recently revealed that intermediate magnetic states can be retained under gradually increased external magnetic field, demonstrating a possibility of making memristors based on the multi-domain switching dynamics (Wang et al., 2019). Further studies are needed for validating reliable read and write schemes in such composite devices. In order to integrate such granular structures into integrated electronic devices, heterostructure of MTJ/granular layer may be used. FL of MTJ can be coupled to the granular layer via interfacial exchange coupling in order to read out the averaged magnetization in the multi-granular layer. As for writing mechanism, heavy metals such as β-W or Ta could be deposited as underlayer next to the granular layer, and thus SOT-driven switching can be exploited in addition to STT approach.

#### 2.2.2. Neuromorphic Devices Based on Exchange-Coupled Heterostructures

**Figure 6:**
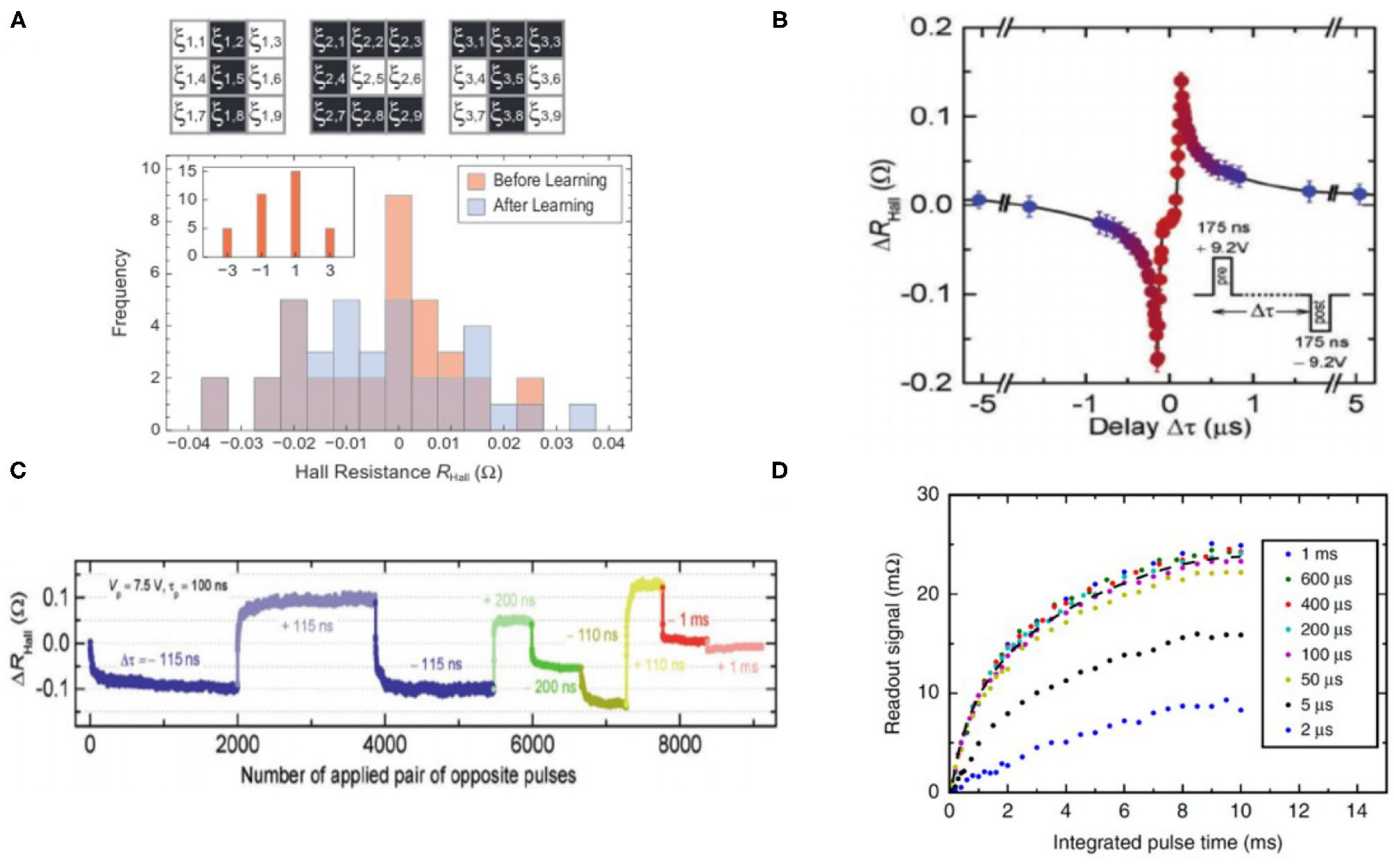
Neuromorphic implementation based on exchange-coupled heterostructures. **(A)** Modifications of synaptic weights stored in F/AF devices after training for pattern recognition. Figure reproduced with permission from Borders et al. Appl. Phys. Express 10 013007 (2017). **(B)** STDP demonstration based on the Hall resistance of F/AF devices. **(C)** Effect of input pulses on the synaptic state of F/AF devices. Figures reproduced with permission from Kurenkov et al. Advanced Materials 31, 1900636 (2019). **(D)** Resistance modifications of an antiferromagnetic material CuMnAs under pulses of various lengths. Figure reproduced from Olejník, K. et al. Nat. Commun. 8, 15434 (2017). Copyright 2017 Author(s), licensed under a Creative Commons Attribution (CC BY) license.

The possibility of inducing multi-states based on antiferromagnetic and exchange-coupled heterostructures ignited growing interest given its potential in developing neuro-inspired devices and hardware primitives. The field is still evolving rapidly today with ongoing efforts in various directions. As for F/AF heterostructures, It is recently shown that the non-volatile analog device build on [Co/Ni]/PtMn can provide synaptic weight matrix of a simple Hopfield Model which can be trained on device and realize associative memory operation as illustrated in Figure 6A (Borders et al., 2016). As is shown in Figures 6B,C, bio-plausible functionalities such as STDP and synaptic plasticity in response to input pulse trains are also demonstrated with the F/AF heterostructure, where STDP were achieved with pre-neuron and post-neuron spikes represented by opposite polarities (Kurenkov et al., 2019). Moreover, AF-only material also demonstrated synaptic behavior in response to accumulated pulses, opening up the possibility of AF-only neuromorphic devices (Figure 6D; Olejník et al., 2017). As for continuous/granular heterostructures, although multilevel magnetic states have demonstrated in CoPt-based composites, device integration into compact memristive prototype with MRAM type of read/write remains to be shown further down the road.

#### 2.2.3. Prospect of Multi-Level Devices Using Exchange Coupled Systems

The leverage of exchange-coupled magnetic systems in achieving multi-level resistive states for neuro-inspired devices brings in new momentum into neuromorphic spintronics. With utilization of fundamental material properties, those multi-level devices based on the non-coherent switching of multi-domains becomes less dependent on special device geometry, which is usually needed for the quasi-1D confinement of a single domain wall. Although both F/AF and ferromagnetic continuous/granular exchange coupled heterostructures hold great promises, critical challenges such as scalability of domains confined by grains and integration with TMR-like readout mechanism will have to be addressed. At present, the resistance change of several Ohms (or less than few percents) in most of recent device-level demonstrations are not feasible for CMOS circuits to sense and process in a integrated chip. Combined efforts of microstructure segregation and antiferromagnetic order on MTJ-based platform could potentially pave the way for providing scalable multi-level spintronic integrated devices for neuro-inspired computing.

## 3. Ferroelectric Devices

Ferroelectric materials can maintain electric polarization states that can be switched by applying voltages (electric fields), and therefore have been under continuous investigations for non-volatile memory applications. With multi-domain switching dynamics taken into consideration, ferroelectric materials are capable of realizing not only as binary synapse but also as multi-level synapse. FeFET and FTJ are the most studied device structures for implementing ferroelectric neuromorphic hardware, and will be discussed in details in the following subsections.

### 3.1. Multi-Level Devices Based on Ferroelectric FET

#### 3.1.1. Material and Device Fundamentals of FeFET

An FeFET integrates a ferroelectric layer into the gate stack of the transistor, generating non-volatile channel conductance of the transistor modulated by the polarization switching in the FE layer. Previously, materials having a perovskites crystal structure such as BaTiO_3_ (BTO) and PbZr_x_Ti_1−x_O_3_ (PZT) are used as ferroelectric materials. In addition to perovskites, novel 2-dimensional (2-D) materials such as transition metal dichalcogenides are being explored in ferroelectric devices (Ko et al., 2016; Zhao et al., 2020). Although FeFETs have been studied extensively given the potential for voltage-controlled non-volatile memory technology, challenges in material integration and scaling have greatly hindered its developments. Interestingly, the recent finding of high coercive field ferroelectricity (Böscke et al., 2011) with low permitivity in doped HfO_2_-based thin films (layer thickness ≤ 20 nm) offers distinctive advantages such as improved retention, scalability, and high CMOS compatibility in comparison to previously investigated materials such as PZT (Gong and Ma, 2016). Therefore, the following discussion of implementing neuromorphic functionalities with FeFETs will focus on HfO_2_-based devices.

**Figure 7:**
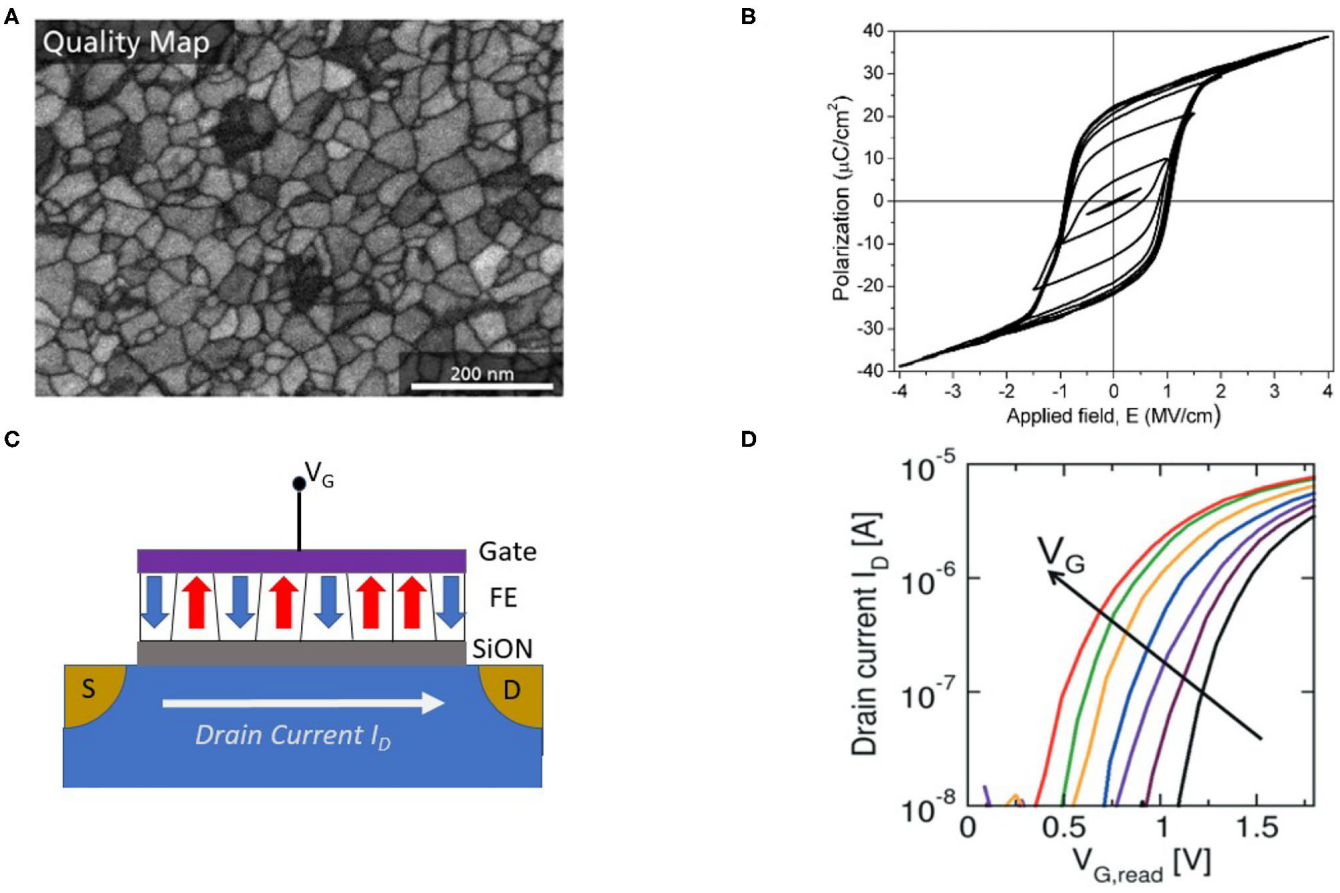
**(A)** Image of polycrystalline multigranular HfZrO_2_ thin film, where average grain size is around 30 nm. Figure reproduced with permission from Lederer et al. Appl. Phys. Lett. 115, 222902 (2019) Copyright 2019 AIP Publishing. **(B)** Polarization-electric field (P-E) loops with hysteresis of HfO_2_. Figure reproduced with permission from Zhou et al. Acta Mater. 99 (2015) 240–246. Copyright 2015 ELSEIVER. **(C)** Device structure of a FeFET. **(D)** Conductance states of FeFET modulated by the gate voltage. Figure reproduced with permission from Mulaosmanovic et al. (2017) Symposium on VLSI Technology (p. T176–T177) Copyright 2017 IEEE.

As for neuro-inspired computing, FeFET devices can produce wide range of electrically controllable non-volatile conductance states which are of particular interest for emulation of neuronal behavior and synaptic plasticity. As is illustrated in Figure 7, intermediate partial polarization states, in addition to the bidirectional fully polarized states, can be induced in FeFET with several FE domains. The multi-domain switching dynamics of the electrical polarization have been observed in polycrystalline thin films of doped HfO_x_ such as Si:HfO_2_, Zr:HfO_2_,or Hf_0.5_Zr_0.5_O_2_ (Zhou et al., 2015; Oh et al., 2017). This polycrystalline film characteristic is determined by post-deposition annealing, which is amorphous without annealing. The normally used annealing temperature range is between 400 and 600°C. After the annealing process, FeFET could have the crystallized ferroelectric layer and this enables FeFETs to have non-volatile functionality by using the remnant polarization. The evolution of multiple ferroelectric domains is captured by polarization-electric field (P-E) hysteresis loops. As is shown in Figure 7B, minor loops of different sizes are generated by varying the ranges of the sweeping electric fields in doped HfO_2_. The non-coherent switching of electric polarization are contributed to the inherent distribution of coercive electric field (E_c_) among the FE grains (Wang et al., 2020). Most of minor loops have non-zero and differentiable remnant polarization states, which provides capability of storing non-volatile multi-bit information in ferroelectric layer. Note that FeFETs are three or four terminal devices, and the multi-level conductances can be characterized by the polarization-dependent threshold voltage, as is shown in Figure 7D (Mulaosmanovic et al., 2017).

#### 3.1.2. FeFET Based Neuromorphic Devices

**Figure 8:**
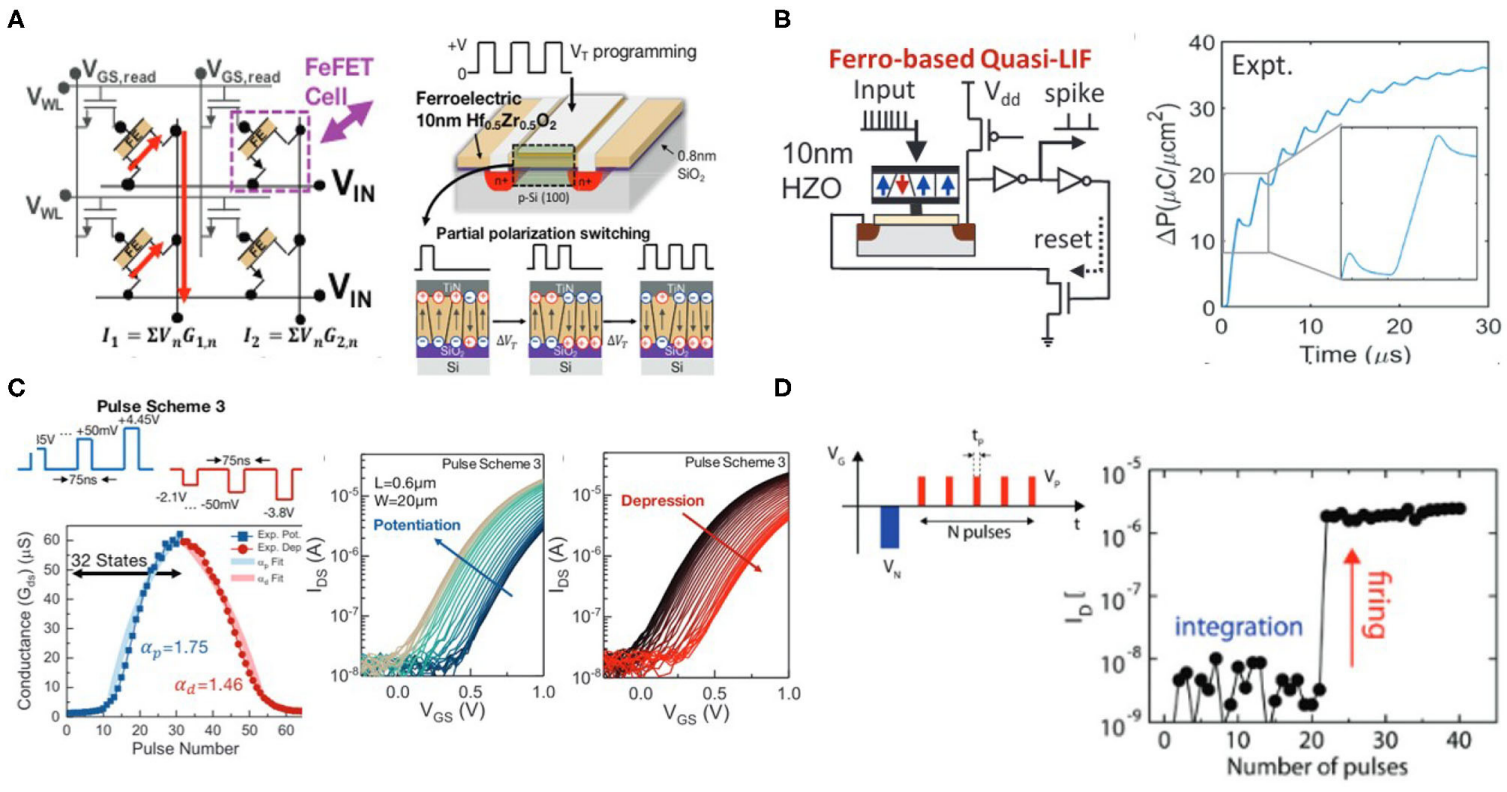
**(A)** Crossbar implementation of multi-level FeFET cells for neuromorphic computing. Figure reproduced with permission from Jerry et al. (2017) IEEE International Electron Devices Meeting (IEDM), p. 6-2, Copyright 2017 IEEE. **(B)** Ferroelectric-based device emulating a leaky-integrate-fire (LIF) neuron. Figure reproduced with permission from Dutta et al. (2019) Symposium on VLSI Technology T140-T141 (IEEE) Copyright 2019 IEEE. **(C)** Voltage pulsing scheme achieving synaptic weight updates in FeFET devices with improved symmetry in conductance change during potentiation and depression processes. Figure reproduced with permission from Jerry et al. In 2017 IEEE International Electron Devices Meeting (IEDM), p. 6-2, Copyright 2017 IEEE. **(D)** FeFET based IF spiking neuron. The firing of neuron can be observed and sensed from the abrupt change of drain current. Figure adapted from Mulaosmanovic, et al. Nanoscale 10, 21755–21763 (2018) with permission from The Royal Society of Chemistry.

The device characteristics of FeFET can be utilized for efficient emulation of both synaptic and neuronal functionalities at the device level. For synapse weight update, there are different pulsing schemes in potentiation and depression and one of the methods incremental programming voltage is widely employed to obtain larger conductance range and symmetry and near-linearly increasing or decreasing conductance. It has been reported that Zr-doped HfO_2_ thin film is capable of demonstrating up to 32 states (5-bit) of channel conductance with an maximum on/off conductance ratio (G_on_/G_off_) of 45 (Jerry et al., 2017). The improved symmetry in weight updates, as shown in Figure 8, could make ferroelectric devices more suited for on-chip training in terms of reliability and predictable synaptic weight compared to training with other memristive memories.

Moreover, neuron behaviors such as input accumulation, leaking in membrane potential, and spike firing can also be emulated utilizing the field-time dependence of domain nucleation kinetics in ferroelectric materials, as shown in Figures 8B,D (Mulaosmanovic et al., 2018; Dutta et al., 2019). In presence of input spike trains as voltages applied across the ferroelectric layer, the portion of switched electric polarization from the multiple domains accumulated, leading to modulation of the output conductance (*G*_*DS*_) and drain current *I*_*DS*_. And leaky behavior due to reduction of the remnant polarization retention has also been observed. Such leak in ferroelectric can be resulted from gate leakage current, effects from interface charges, oxide breakdown, intrinsic depolarizing field in a ferroelectric capacitor, or a reversed inhibitory electric field. It was also observed that abrupt change in channel conductance can take place under scaled devices which have just few grains so that each grain switching effect are largely reflected into the conductance change, effectively emulating a step-like integrate-fire neuron. The sharp transition from OFF to ON state is attributed to formation of conductance pathway in the channel when a sufficient number of subdomains have switched.

#### 3.1.3. Prospect of Multi-Level FeFET

FeFET with HfO_2_-based thin films has shown great potential toward the development of non-volatile neuromorphic devices. The capability of emulating both synapse and neuron functionalities with the same device structure could provide an advantage toward hardware integration. More importantly, the ferroelectric material has high CMOS compatibility, while the possibility of realizing symmetric conductance modulation, as well as demonstrated high on/off ratio makes it competitive for implementing both training and inference machine learning tasks at large scales. Meanwhile, some critical issues still remains. In general, HfO_2_-based FeFETs still have endurance and retention issues associated with the interfacial layer deposited between ferroelectric and channel. This is because the degradation in remnant polarization due to the impact of charge traps, defects, and oxide breakdown. Currently the endurance of HfO_2_-based FeFET is about 10^9^ cycles (Oh et al., 2019), still requiring significant improvements for industrial applications. A paramount challenge for using FeFET as neuromorphic multi-level devices is the scaling limit of the device dimension. Multiple conductance states, a device prerequisite for emulating synaptic plasticity, are determined by intermediate partial polarization states. At present it is found that the average domain size of Zr-doped HfO_2_ thin film is about 30 nm (Lederer et al., 2019), which is significantly smaller than domains in conventional PZT materials, but is still quite large compared to the state-of-art CMOS feature size. Therefore, more exploratory device and material engineering, such as device structure, doping and post-annealing temperature optimization, may be needed to maintain multi-level polarization states while further pushing down the devices dimensions.

### 3.2. Multi-Level Ferroelectric Tunnel Junctions

#### 3.2.1. Device Fundamentals of FTJ

**Figure 9:**
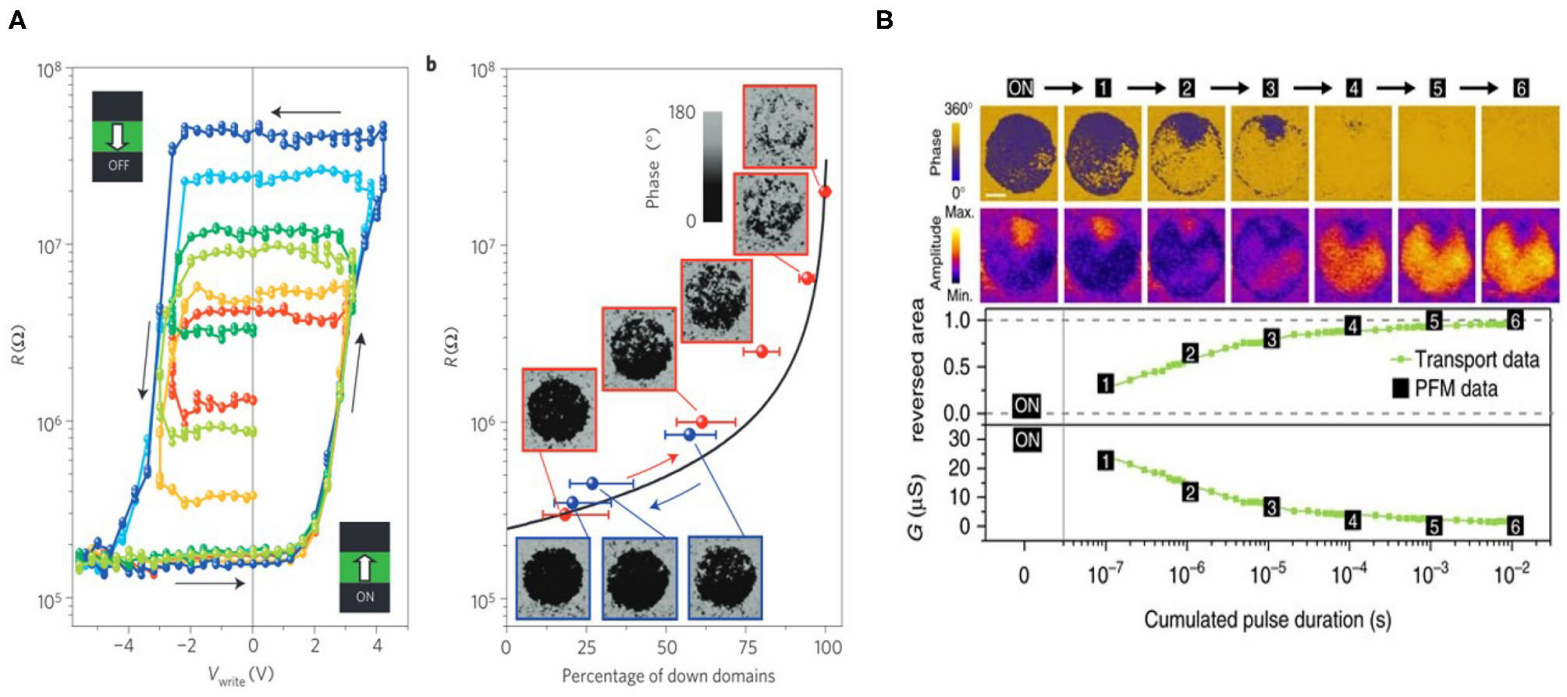
**(A)** Multi-level state characterized by tunneling electro-resistance of a FTJ comprising BaTiO_3_ as the tunnel barrier sandwiched between Au/Co and LSMO electrodes. The resistance states are found to follow the percentage of domains switched by applied voltages. Figure reproduced with permission from Chanthbouala et al. Nat. Mater. 11, 860–864 (2012). Copyright 2012 Springer Nature. **(B)** Gradual conductance change of a BaFeO_3_ based FTJ under accumulated pulses is experimentally observed to be closely associated with the switching process of ferroelectric multi-domains. The behavior of conductance change under pulses can be directly implemented into neuromorphic functionalities such as synaptic plasticity. Figure reproduced from Boyn et al. Nat. Commun. 8, 14736 (2017). Copyright 2017, Authors, licensed under a Creative Commons Attribution (CC BY) license.

Another subclass of multi-domain devices built on ferroelectrics is ferroelectric tunnel junctions (FTJs). FTJs are two terminal devices with a layer of ferroelectric material sandwiched between top and bottom metallic layers of electrodes, where sizeable tunneling currents can occur at room temperature if the ferroelectric layer is as thin as few nanometers. Although the concept of FTJ has been proposed in 1971 (Esaki et al., 1971), device realization has been challenging due to the fact that ferroelectricity are vanishing with decreasing film thickness, while tunneling junctions need to work with thin tunnel barriers. FTJ started to gain considerable interest from the 2000s when high quality ultrathin ferroelectric films (thickness ≤ 10 nm) were made possible by epitaxial deposition. Heterostructures of complex oxides based junctions are the most widely studied FTJ systems. With the ferroelectric barrier layer sandwiched between different electrode materials, different screening lengths in electrodes break the symmetry of electrostatic potential profile across the FTJ tunnel barrier. Such electrostatic potential profile will be modified by the reversal of ferroelectric polarization, leading to considerable tunneling resistance variation known as the tunneling electro-resistance (TER) effect. FTJs made with various ferroelectric materials including BaTiO_3_ (BTO), BiFeO_3_ (BFO), and Hf_0.5_Zr_0.5_O_2_ (HZO) have demonstrated large range of modulation in the tunneling electroresistance (with typical G_ON_/G_OFF_ Ratio of 10–10^4^) (Chanthbouala et al., 2012; Ambriz-Vargas et al., 2017; Boyn et al., 2017), suggesting a promising pathway for non-volatile memory applications. Similarly to the scenario in FeFET, the polarization switching in ferroelectric ultrathin films involves domain nucleation and propagation dynamics, providing exciting opportunities for emulating memristive neuromorphic functionalities with electric control. As is shown in Figure 9, resistance levels in perovskite-based FTJs are modulated following the portion of domain being switched, confirming the multi-domain nature behind the memristive behavior.

#### 3.2.2. FTJ-Based Neuromorphic Devices

**Figure 10:**
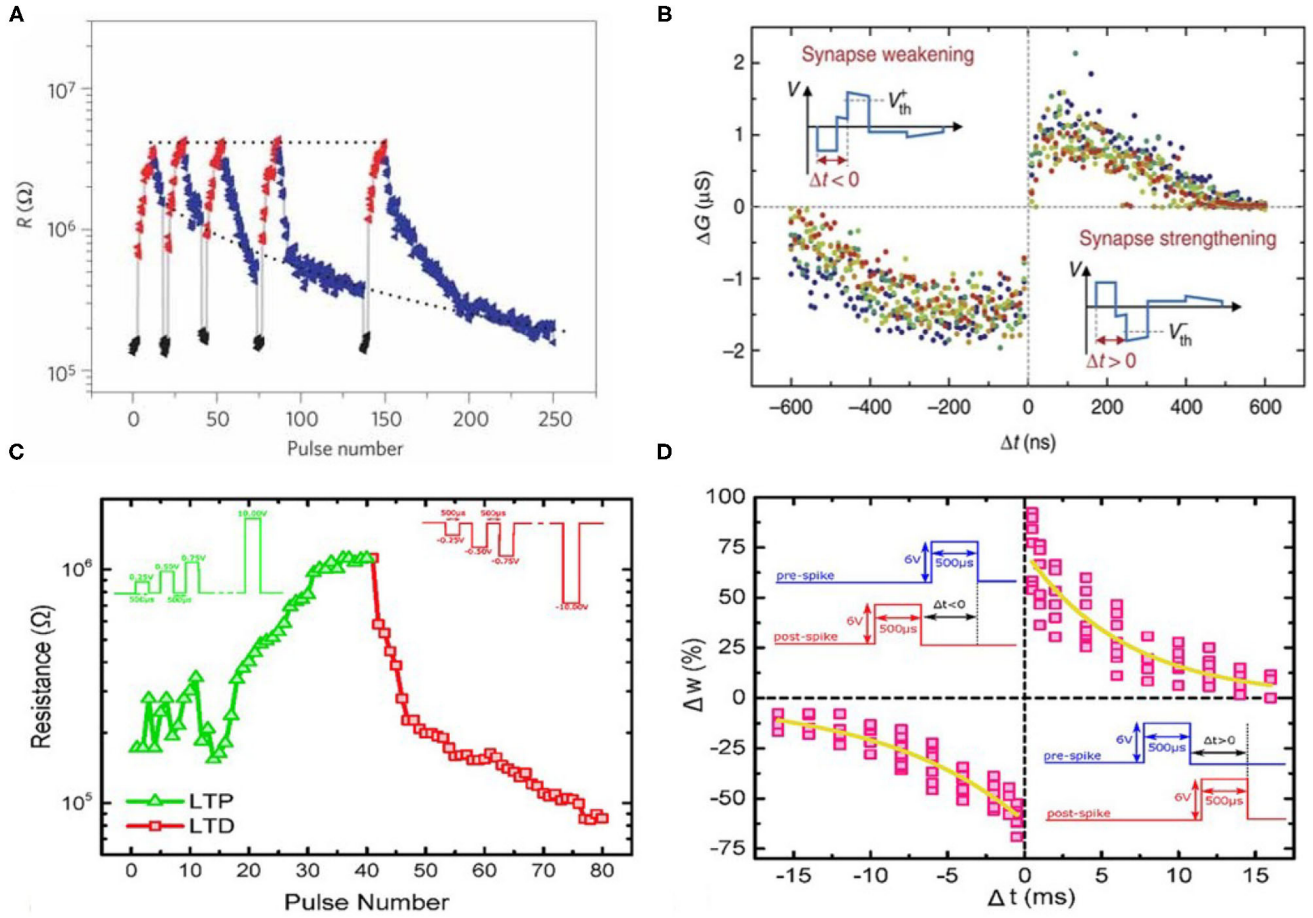
**(A)** Demonstration of synaptic potentiation and depression in a BaTiO_3_-based FTJ. Figure reproduced with permission from Chanthbouala et al. Nat. Mat. 11, 860–864 (2012) Copyright 2012 Springer Nature. **(B)** STDP demonstration in BaFeO_3_ based FTJ. Figure reproduced from Boyn et al. Nat. Comm. 8, 14736 (2017). Copyright 2017, Authors, licensed under a Creative Commons Attribution (CC BY) license. **(C)** Demonstration of synaptic potentiation and depression and **(D)** STDP in HfO_x_ based FTJ. Figures reproduced with permission from Yoong et al. Adv. Funct. Mater. 2018, 28, 1806037. Copyright 2018 WILEY-VCH Verlag GmbH & Co. KGaA, Weinherm.

Based on the dynamics of multi-domain nucleation, a fine tuning in tunneling resistance state is demonstrated corresponding to a gradually increased portion of switched ferroelectric domains under incremental voltage or repetitive series of constant voltage signals. As is shown in Figure 9B, the pulse duration dependence of the gradual domain evolution in BFO-based FTJ suggests that FTJs can emulate highly bio-plausible behavior utilizing the multi-domain switching dynamics. Long term potentiation and depression (LTP/LTD) under positive and negative voltage pulses (as illustrated in Figure 10A) have also be demonstrated, paving the way toward practical implementation of synaptic plasticity for brain-inspired hardware primitives (Chanthbouala et al., 2012). Moreover, STDP can also be achieved with device conductance modulation as a function of delay between pre-synaptic and post-synaptic spikes, as is shown in Figure 10B (Boyn et al., 2017). In addition, it is encouraging to see that similar investigations of realizing neuromorphic functionalities such as synapse potentiation/depression (Figure 10C) and STDP (Figure 10D) were also recently achieved with Hf_0.5_Zr_0.5_O_2_ (Yoong et al., 2018), suggesting a viable path of fabricating FTJs with good CMOS compatibility.

#### 3.2.3. Prospect of Multi-Level FTJ

Given the advantages of relatively simple device structure with controllable conduction mechanism, high ON/OFF ratio, fast switching and non-destructive multi-level dynamics, FTJ holds a promising prospect for implementing synaptic devices. In particular, FTJ as a two-terminal device can be better suited for crossbar implementations of synaptic weight matrices. But FTJ-based synapses are still in early research stage with several issues to address. One major issue is that most FTJs require high voltages (⩾ 2–5 V) applied over the tunnel barrier to switch the polarization, making it challenging for scalable circuit integration. One of the approach to reduce the operation voltage is thinning ferroelectric tunnel barriers, however, the retention and stability of polarization is deteriorated, limiting retention of current FTJs on the order of 10–100 h (Guo et al., 2020). Another generic challenge is the scalability of ferroelectric multidomains as is also faced by FeFET devices. Moreover, phenomena associated with polarization reversals, such as interfacial charge trapping and ion migrations could induce defects that negatively impact on device endurance, and thus most FTJ devices to date have endurance of no larger than 10^6^ cycles (Guo et al., 2020). And the shared path of TER reading and polarization switching could further lead to additional reliability issue such as destructive read. Another challenge is that tunneling current is exponentially depending on the thickness of the tunnel barrier so that precise thickness controlling is needed. From the viewpoint of practical manufacturability, more explorations are needed as most FTJs to date rely on exotic ferroelectric materials such as perovskites and/or unconventional electrode materials due to material growth constraints. In this respect, HfO_x_-based FTJs are considered as an emerging candidate given the less stringent condition of synthesis and Si CMOS process compatibility.

## 4. Discussion

The multi-level device characteristics utilizing collective multi-domain dynamics of magnetization or electric polarization switching have successfully demonstrated device-level emulation of neural functionalities and could be leveraged to build robust and energy efficient bio-plausible hardware primitives for AI applications.

Spintronic and ferroelectric devices could potentially provide some advantages compared with ReRAM/PCM and CMOS technologies. The spin-based devices require a lower programming voltage compared to ReRAM (Park et al., 2013; Adam et al., 2017) and PCM (Papandreou et al., 2011; Tuma et al., 2016). They also demonstrate higher endurance compared to ReRAM and PCM (Prenat et al., 2016; Li et al., 2019). While the multi-bit capability of spintronic devices may be limited by the low ON/OFF ratio (G_On_/G_Off_ ~ 2–3), it is worthwhile to note that high precision weight matrices can be mapped to multiple crossbars in large scale implementation of in-memory computing. Moreover, multi-level cells having large number of states would typically require a higher precision readout circuitry to interface between digital and analog domains, dominating the power and area costs (Shafiee et al., 2016; Ankit et al., 2019). On the other hand, FeFET and FTJ could provide large dynamic ranges (G_On_/G_Off_ ≤ 10^2^) with numerous intermediate states. Hence, multi-level spintronic devices can be well suited for implementing frequently updated components such as neurons, while ferroelectric devices are considered to be more suitable for implementing analog synapses, given the device characteristics of large memory windows between states, low read/write energy, fast switching, and superior CMOS compatibility (Khan et al., 2020). In the following, we will elaborate particular challenges of implementing crossbar in-memory computing based on ReRAM and PCM materials, and highlight the strength in spintronic and ferroelectric device characteristics that could potentially address some of the challenges. A near-term scenario of NVM based neuromorphic computing is to execute AI inference tasks with pre-trained models mapped into crossbar arrays, while a more challenging scenario is to enable on-chip learning.

As for the inference-only scenario, the crossbars are in read-only mode. The MVM operations will be executed based on the product of the input voltage and the weight matrix, which are stored as device conductances. In general, it is desirable to have large storage density of devices with distinctive states and strong data retention against thermal agitation and other relaxation mechanisms over an extendable time. PCM based on alloys such as Ge_2_Sb_2_Te_5_, although capable of high density with large dynamic range (G_on_/G_off_ ~ 10^3^), suffers resistance drift due to relaxation of the amorphous state (high resistance states). Such drift in device conductance significantly degrades the desirable data retention and thus requires additional circuit-level compensation scheme in real applications, leading to additional energy consumption and delay (Yu and Chen, 2016). As for filament-based ReRAM with oxides such as HfO_x_/TaO_x_, although it has advantages of compact cell/array size, large device variability (especially at high resistance states) can be a major hindrance (Yu and Chen, 2016; Li et al., 2019). The large device variation not only places challenges on the sensing circuit but also leads to a reduced number of bits per cell, even when the device-level conductance ON/OFF ratio is high (Chakraborty et al., 2020b). On the other hand, the memory effect of ferroic (ferromagnetic or ferroelectric) orderings are well-poised given their advantages in storing information with superior retention. In particular, spintronic devices can store information based on the magnetizations in materials with strong anisotropy. The thermal stability of the bits stored in spintronic devices is governed by ratio of the energy barrier of switching over thermal fluctuation Δ = *E*_b_/*k*_B_*T*, where E_b_ is the effective energy barrier of magnetization switching, k_B_ is the Boltzmann constant and T is the operation temperature. With Δ ~ 60–80 in current spintronic devices, superior data retention has been demonstrated on the order of years in memory and data storage applications. In addition, the fact that electro-forming is overall a one-dimensional process suggests that device and cycle variations could always be an issue with filament-based device such as ReRAM. Note, multi-domain devices are fundamentally not limited to 1D process and in principle, can be modeled as parallel conduction channels. Such conceptually parallel channels can be less prone to variations, thanks to the averaging effects of the collective channels. Therefore, both spintronic and ferroelectric devices suffer less from device variability compared to filament based memristive technologies. Another pressing challenge of running MVM on crossbar arrays is the non-ideality associated with crossbar circuits. While large crossbars are desirable to utilize the massive parallelism in crossbar based MVM, the non-ideality due to voltage drop along wire resistance and other circuitry components (“IR” drop) will become more severe at larger crossbars, leading to non-ideal output current values compared to the ideal crossbar output current I = G · V. Note that using devices with low ON-state resistance will be more susceptible to the non-ideal IR drop, and thus a high ON-state resistance is desirable for accurately performing crossbar-based inference task (Chakraborty et al., 2020a). PCM and ReRAM typically have ON-resistance of 10–100 kΩ, while FeFET can operate at ON-resistance of higher than 500 kΩ (Jerry et al., 2017). Emerging spintronic devices such as spin orbit torque (SOT)-MTJ can potentially work under MΩ ON-resistance, due to separated read/write paths (Doevenspeck et al., 2020). The ability of providing large ON-state resistance can potentially enable deployment of large crossbars with minimal impact on computing accuracy.

On-chip learning and training using crossbar arrays of memristive devices are even more challenging, since both read and write operations will be involved and thus the device conductance in crossbar arrays will be re-written frequently throughout training. One major issue of both ReRAM and PCM is the limited endurance (10^6^–10^8^ write cycles). The chalcogenide alloy in PCM experiences frequent expansion and contraction under heating during writing process, and the high probability of physical detachment at the interface between the alloy and the heating elements can lead to permanent defects in devices, leading to hard errors such as stuck at fault when device states are no longer changeable. As for the ReRAM, both intrinsic device variation originated from fabrication process and the writing/resetting of ReRAM devices can contribute to severe device defects that can significantly degrade the accuracy of the crossbar in-memory computing. In contrast, the endurance of ferromagnetic devices is much better in comparison with ReRAM and PCM, since switching in spin-based devices do not need electro-forming of conduction filaments (or forming conductive phase through heat-induced crystallization). The endurance of production-ready STT-MRAM can be as high as 10^14^–10^15^ cycles with write latency of 10 ns, making MRAM much more competitive for data memory requiring frequent updates. And newly emerging SOT-MTJ equipped with separated read and write current paths could provide even more flexibility in design fast and high-endurance devices. The leverage of MRAM technology for neuromorphic computing can provide a viable path toward making crossbar arrays with high endurance for training and learning. Moreover, writing in PCM and ReRAM requires high energy consumption and latency due to the device characteristics. The phase transition in PCM needs substantial write current (~100 μA for a 20 nm device as reported in Kang et al., 2011) to heat and melt the material, and the switching speed is limited by the relatively slow crystallization process (>50 ns; Yu and Chen, 2016). As for the filament-based ReRAM materials, large device-to-device and cycle-to-cycle variability resulting from the stochastic nature of ion migration and variation of the filament shape and structure have always been a critical issue for training in ReRAM devices. Although single write of a ReRAM device can be fast and efficient, multiple write-verify cycles are needed to set a device into a desirable conductance level, effectively raising the cost of writing. Therefore, training directly on arrays of PCM/ReRAM devices can lead to high energy consumption and significant delay as well as degradation in computing accuracy due to device failure. The high cost of writing with ReRAM and PCM crossbars can be potentially mitigated by using devices based on spintronic and ferroelectric materials. Changes of device states in ferromagnetic and ferroelectric materials rely on magnetization or polarization switching, which can be more energy efficient since no crystallization/melting process or significant ionic motion are involved. Note that utilizing the multi-domain switching dynamics provides a statistical averaging effect over the multiple domains involved, reducing the device and cycle variations. In addition, the implementations of neurons, compared to synapses, typically have more relaxed requirement of density, but may have more stringent requirement on endurance due to the possibly frequent occurrence of activation. At present, most of the available ReRAM and PCM materials do not have the desirable endurance to execute neuron activation. Therefore, we believe that emerging ferroic devices and systems with superior endurance can have exciting opportunities in providing non-volatile neuronal devices in addition to synapses. Such implementation could potentially lead to significant improvement in energy efficiency due to reduced data movement between memory and neuron units.

In spite of the tremendous progress in implementing synaptic as well as neuronal functionalities based on a plethora of multi-domain materials, further explorations are still needed to tackle several key challenges. First of all, large device footprints are typically desirable for demonstrating multi-domain switching mechanism in either spintronic or ferroelectric devices, limiting the synaptic memory density that can be implemented. In magnetic devices, multi-domain switching are found to vanish in ferromagnets as the device size decreased to 200 nm, even in presence of interlayer coupling to adjacent multi-domain antiferromagnets (Kurenkov et al., 2019). Therefore, achieving energy-efficient generation and control of multi-domains in a scalable spin-based material/device platform still requires further explorations. In ferroelectric devices, most demonstrations of memristive behaviors to date are done on devices with lateral size of 200 nm to few microns, but it remains to be seen if the multi-level polarization switching can still persist as devices dimension shrinks to the size comparable to size of a nucleation site in ferroelectric thin films. Moreover, the spintronic and ferroelectric technologies also have their own challenges to address. As for spintronic devices, continuous efforts are needed for smooth integration of multi-domain functional blocks with manufacturable and programmable devices such as MTJs. In particular, how to utilize novel mechanisms such as voltage-controlled magnetic switching, spin-orbit torques in emerging materials such as antiferromagnets, multiferroics, or granular composite structures for generating multi-level in a scalable integrated devices will be of significant interest. Magnetic devices are further challenged by limited readout resolutions due to the relatively small magneto-resistance effect in practical MTJs. Fortunately, emerging SOT-MTJ with separate read and write paths could lead to device optimized in new design space with possibly higher ON/OFF ratios (up to 6x larger TMR ratio) that could be better suited for neuromorphic computing (Ikeda et al., 2008; Doevenspeck et al., 2020). And novel materials such as half metal have been predicted to potentially boost the magnetoresistive signals in future (Bhatti et al., 2017). In devices built on ferroelectric thin films (thickness <10 nm), growing depolarizing effects and charge redistribution/trap at interfaces across the heterostructures significantly limit the retention and endurance of the remnant polarization states. Therefore, further investigations of materials and device physics will be crucial in achieving reliable and scalable multi-domain neuro-inspired hardware primitives toward sub-10-nm domains.

**Figure 11:**
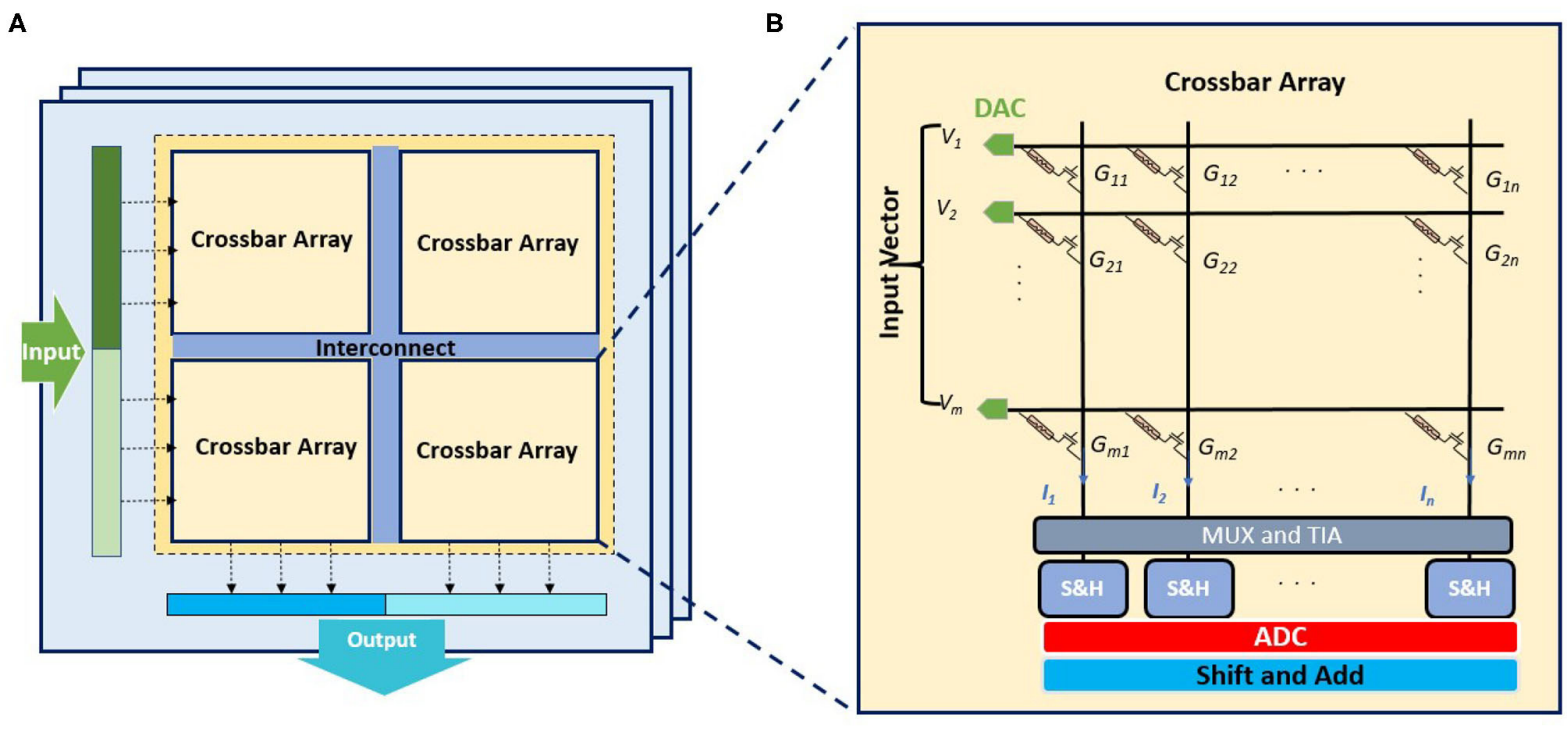
In-memory computing architecture involving multiple tiles of interconnected crossbar arrays to process high dimensional MVM operations with high bit precision. **(A)** Illustration of the multi-tile architecture for executing large input vector with high bit precision where multiple crossbars will be used for mapping the input and weight matrix. **(B)** Illustration of the circuit of a single crossbar array comprising the array of synaptic devices in series with access transistors and peripheral analog and digital circuits. A multiplexer (MUX) and transimpediance amplifier (TIA) are directly connected to the crossbar columns, followed by sample and hold circuit (S&H) as well as ADC and shift and add circuitry.

The multi-level spintronic and ferroelectric devices, which enable MVM acceleration and efficient emulation of neural functionalities, provide building blocks for implementing large-scale computing system. Note that complex AI tasks such as image classifications or natural language processing usually need to run large DNN models comprising multiple layers, where the size of involved matrices can be significantly larger than typical crossbar sizes. Furthermore, in order to yield satisfactory prediction accuracy, the model parameters in AI algorithms such as DNN will need higher resolution (for example, 16–32 bit synaptic weights) compared to the highest possible bits per cell in synaptic devices (about 1–5). Therefore, the mapping of input vectors and weight matrices into hardware may involve multiple arrays or multiple columns (Shafiee et al., 2016). The bits of input vector are converted to voltages, and they can be streamed in time depending on the bit-precision of the digital-analog converters (DAC) (Ankit et al., 2019). The accumulated current in crossbar column obtained from the dot product of the input voltage vector and conductance matrix will be processed in combination with results from multiple time steps (bit streaming) and/or multiple columns/arrays (bit slicing) to execute multi-bit MVM operations. In order to minimize noise accumulation through these aforementioned multi-bit operations, digital circuitry such as shift and adder are needed, requiring the conversion of analog signals to digital domains via analog-digital converters (ADC). Moreover, processing of partial sums from multiple crossbar arrays are needed in the case of large matrices for generating the final outputs, which also desire ADCs in order to leverage the noise resilience of digital circuitry. As is shown in Figure 11, crossbar arrays of multi-level devices provide building blocks for partitioning and mapping of a neural network model, so that workflows involving large and high precision models can still leverage the advantage of the underlying device characteristics of non-volatile multi-level memory. Crossbar arrays of devices with large number of bits per device will increase the requirement of ADC bit precision, leading to significant increase of power and area costs dominated by the ADC (Shafiee et al., 2016). To this effect, the footprint and energy cost of crossbar array itself may not be the bottleneck. While more crossbars will be needed to map the algorithmic models using devices with fewer bits per cell, better energy efficiency could be reached with low-precision ADCs. Therefore, depending on which factor dominates the system-level performance, a trade-off analysis could lead to optimized designs with different crossbar and ADC configurations. The impacts of device/circuit level settings such as device conductance range, crossbar size, number of states per cell have been demonstrated on the performance of implementing high level AI algorithms (Chakraborty et al., 2020a), while algorithmic characteristics such as sparsity in model parameters can also influence the choice of setting and operation of the crossbar arrays. Therefore, systematic trade-off analysis and application specific co-design across the hierarchy of material, device, circuit, and algorithm will be crucial toward the optimization of system-level performance, and we hope that this review will inspire more research efforts on utilizing the emerging ferroic device technologies toward developing efficient neuro-inspired computing system comprising both hardware and software.

## Author Contributions

All authors listed have made a substantial, direct and intellectual contribution to the work, and approved it for publication.

## Conflict of Interest

The authors declare that the research was conducted in the absence of any commercial or financial relationships that could be construed as a potential conflict of interest.
